# Vitamin B_12_ Deficiency Induces Imbalance in Melanocytes Homeostasis—A Cellular Basis of Hypocobalaminemia Pigmentary Manifestations

**DOI:** 10.3390/ijms19092845

**Published:** 2018-09-19

**Authors:** Zuzanna Rzepka, Michalina Respondek, Jakub Rok, Artur Beberok, Keith ó Proinsias, Dorota Gryko, Dorota Wrześniok

**Affiliations:** 1 Department of Pharmaceutical Chemistry, School of Pharmacy with the Division of Laboratory Medicine in Sosnowiec, Medical University of Silesia in Katowice, Jagiellońska 4, 41-200 Sosnowiec, Poland; zuzanna.rzepka@med.sum.edu.pl (Z.R.); michalina.respondek@med.sum.edu.pl (M.R.); jrok@sum.edu.pl (J.R.); 2 Institute of Organic Chemistry, Polish Academy of Science, Kasprzaka 44/52, 01-224 Warsaw, Poland; keithoproinsias@gmail.com (K.ó.P.); dorota.gryko@icho.edu.pl (D.G.)

**Keywords:** vitamin B_12_, cobalamin deficiency, hyperpigmentations, melanogenesis, oxidative stress

## Abstract

Vitamin B_12_ deficiency causes significant changes in cellular metabolism leading to various clinical symptoms, such as hematological, psychiatric, and neurological disorders. We hypothesize that skin pigmentation disorders may be a diagnostically important manifestation of vitamin B_12_ deficiency, however the cellular and molecular mechanisms underlying these effects remain unknown. The aim of this study was to examine the effect of vitamin B_12_ deficiency on melanocytes homeostasis. Hypocobalaminemia in vitro model was developed by treating epidermal melanocytes with synthesized vitamin B_12_ antagonist—hydroxycobalamin(*c*-lactam). The cells were examined using immunoenzymatic, spectrophotometric, and fluorimetric assays as well as image cytometry. Significant melanogenesis stimulation—the increase of relative melanin content and tyrosinase activity up to 131% and 135%, respectively—has been indicated. Cobalamin-deficient cells displayed the elevation (by 120%) in reactive oxygen species level. Moreover, the redox status imbalance was stated. The study provided a scientific evidence for melanocytes homeostasis disturbance under hypocobalaminemia, thus indicating a significant element of the hyperpigmentation mechanism due to vitamin B_12_ deficiency. Furthermore, the implication between pigmentary and hematological and/or neuropsychiatric symptoms in cobalamin-deficient patients may be an important issue.

## 1. Introduction

Vitamin B_12_ (cobalamin) deficiency is an important health issue, particularly among the elderly. The main causes of hypocobalaminemia are insufficient dietary intake (e.g., in vegans and vegetarians) and malabsorption of the vitamin [1]. The nearly ubiquitous use of drugs that can interrupt cobalamin absorbtion, i.e., gastric acid–blocking agents and metformin, may also contribute to a growing prevalence of cobalamin deficiency [2].

Vitamin B_12_ plays a key role in cellular metabolism. It is a cofactor of two enzymes: Methionine synthase, which catalyzes a methylation of homocysteine to methionine with simultaneous reconstitution of tetrahydrofolate and methylmalonyl-CoA mutase, which catalyzes the conversion of methylmalonyl-CoA to succinyl-CoA. At the cellular level, cobalamin deficiency results in the inhibition of purine synthesis and in the accumulation of methylmalonic acid and homocysteine (Hcy) [1]. Hcy has been previously demonstrated to act as a prooxidant in various types of cells [3,4].

Melanin is a natural pigment and the major determinant of skin and hair color. Biosynthesis of melanin takes place in melanocytes—dendritic cells located mainly in the basal layer of the epidermis and hair follicles [5,6]. The ability to synthesize melanin plays an important role in skin physiology and pathology, and determines skin responsiveness to ultraviolet radiation. Melanogenesis is a complex process controlled by different modes including receptor-mediated pathways activated by hormones, cytokines, neurotransmitters, and eicosanoids, as well as receptor-independent mechanisms modulated by nutrients, microelements, pH, ions, and redox homeostasis [5,7].

The major symptoms of cobalamin deficiency are hematological, psychiatric, and neurological disorders [8]. Less known manifestations include reversible cutaneous hyperpigmentation, mostly localized in the dorsum of hands and feet, fingers, knees, lateral surfaces of the legs, skin folds, and oral mucosa [9,10,11,12]. Interestingly, several clinical cases of vitiligo and depigmentation of scalp hair due to cobalamin depletion have also been reported [13,14]. Thus, the impact of cobalamin deficiency on melanogenesis and melanocytes homeostasis is not obvious.

Current scientific findings about the molecular patomechanism of hypocobalaminemia-induced disorders are limited by the lack of a specific experimental system. The in vivo model of vitamin B_12_ deficiency is difficult to prepare because of pre-existing tissue cobalamin stores in experimental animals [15,16]. Therefore, cell culture systems might be useful for elucidating mechanisms of various disorders induced by hypocobalaminemia. However, under standard conditions, in which the growth media are supplemented with serum, the deficiency cannot be achieved by simple means as serum contains protein-bound vitamin B_12_. This problem may be overcome by cell culturing in the presence of vitamin B_12_ antagonists, e.g., analogue of hydroxycobalamin: (OH)Cbl(*c*-lactam). According to the nomenclature of cobalamin derivatives, (OH)Cbl(*c*-lactam) is the analogue with a modification of the amide group present at the *c*-position of B pyrrolic ring [17].

It has been shown that melanogenesis process and homeostasis in normal melanocytes may be modulated by various agents, e.g., fluoroquinolones [18,19], aminoglycoside antibiotics [20,21,22], tetracyclines [23,24], and nicotine [25]. Recently, it has been demonstrated that melatoninergic system, with its final product, can both have anti-oxidative and anti-melanogenic properties [26].

The aim of this study was to develop an in vitro model for specific investigation of the effect of vitamin B_12_ deficiency on melanin-producing cells, thus providing a scientific explanation of the hitherto unknown cellular and molecular mechanisms of hypocobalaminemia pigmentary manifestations.

## 2. Results

### 2.1. Synthesis and Identification of (OH)Cbl(c-lactam)

**Figure 1:**
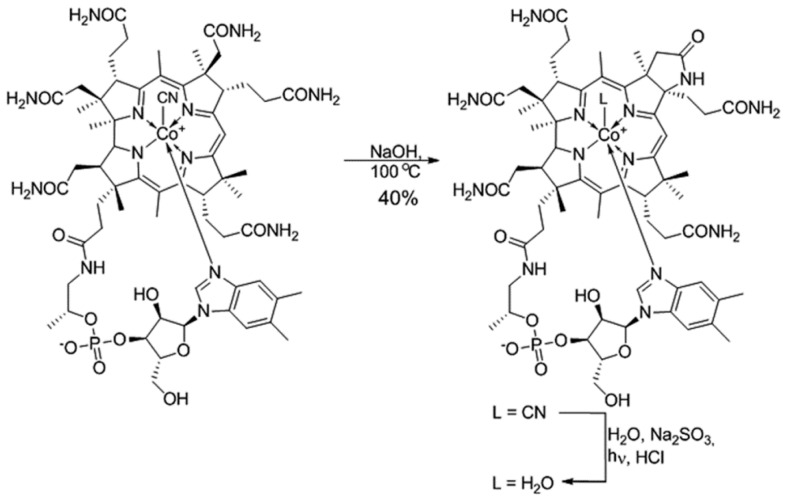
Synthesis of (OH)Cbl(*c*-lactam).

(CN)Cbl(*c*-lactam) was prepared from commercially available (CN)Cbl(cyanocobalamin) according to the literature procedure described by Bonnet et al. [27]. Subsequently, it was transformed into aqua derivative in the presence of Na_2_SO_3_ under light irradiation (Figure 1).

### 2.2. Effect of (OH)Cbl(c-lactam) on Cell Growth

**Figure 2:**
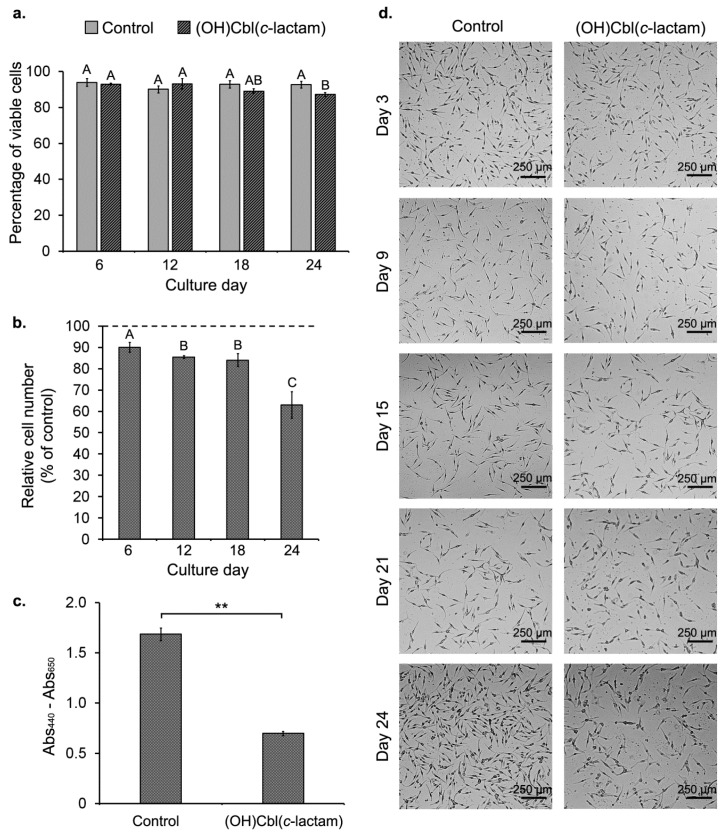
Effect of long-term incubation with (OH)Cbl(*c*-lactam) on melanocytes viability and proliferation. HEMn-DP cells were cultured in medium supplemented with (OH)Cbl(*c*-lactam) in concentration of 10 μg/mL for 24 days. Control melanocytes were cultured in parallel in standard growth medium. (**a**) The percentage of viable cells in total cell population for both treated and control culture was determined at days: 6, 12, 18, and 24 using the image cytometer. (**b**) Total cell number was evaluated at the indicated time points of long-term incubation with (OH)Cbl(*c*-lactam) and these values were expressed as a percent of the values obtained for control cultures (control was set to 100%, indicated by dashed line). (**c**) Data of WST-1 assay performed after 24 days of treatment with (OH)Cbl(*c*-lactam); data were compared to those obtained from control cells. (**a**–**c**) Data presented as the mean values of three independent experiments ± SD; means sharing the same letter are not significantly different at α= 0.05 (ANOVA followed by Tukey’s test, Figure 2a: letter A for control); asterisks denote significant differences from the control (** *p* < 0.01; Student’s *t* test). (**d**) Representative microscope images of melanocytes obtained at the indicated time points of the experiment; cells were observed using light inverted microscope with magnifications of 40×, scale bar = 250 μm.

To achieve an experimental melanocytes-based model of cobalamin deficiency, HEMn-DP cells were cultured in growth medium supplemented with (OH)Cbl(*c*-lactam) in concentration of 10 μg/mL. During sub-culturing, cell count, and viability were determined using NucleoCounter^®^ NC-3000™, as described in Section 4.5. The results showed that long-term exposure to the agent only slightly affected the viability of melanocytes (Figure 2a). However, compared to the control, (OH)Cbl(*c*-lactam) was found to significantly inhibit the proliferation of melanocytes in a time-dependent manner. As demonstrated in Figure 2b, at day 24 of the treatment, the number of cells was reduced to 63 ± 6% of that in the control culture. In the microscopic observations by inverted microscope Eclipse TS-100-F (Nikon, Tokyo, Japan), the notable decrease in the number of melanocytes cultured with the cobalamin antagonist was reported only at day 24 (Figure 2d). In addition, WST-1 assay performed after 24 days of the incubation revealed a reduction in the number of metabolically active cells by about 60% compared to the control (Figure 2c), thus confirming that long-term treatment of melanocytes with (OH)Cbl(*c*-lactam) significantly suppressed cell proliferation.

### 2.3. Changes in Extracellular Hcy Concentration during Culture with (OH)Cbl(c-lactam)

**Figure 3:**
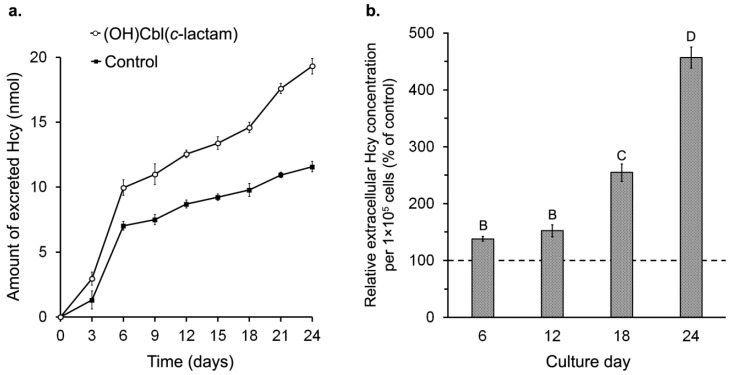
Homocysteine (Hcy) accumulation in culture medium during long-term incubation of melanocytes with (OH)Cbl(*c*-lactam). (**a**) The cumulative amount of Hcy excreted my melanocytes at the indicated time points of long-term incubation with the cobalamin antagonist; data were compared to those obtained from control cells. (**b**) The Hcy concentrations in medium collected at days 6, 12, 18, and 24; the results were normalized to 1 × 10^5^ cells and expressed as a percent of the values obtained for control (control was set to 100%, indicated by dashed line). Data presented as the mean values of three independent experiments ± SD; means sharing the same letter are not significantly different at α = 0.05 (ANOVA followed by Tukey’s test, letter A for control).

To determine a time after which melanocytes cultured with (OH)Cbl(*c*-lactam) are cobalamin deficient, we measured concentration of Hcy, well known biomarker of vitamin B_12_ deficiency, in the culture medium samples collected at three-days intervals. Our results revealed that exposure of HEMn-DP cells to 10 μg/mL (OH)Cbl(*c*-lactam) induced successively increasing extracellular Hcy concentration during 24 days in culture (Figure 3a,b). We observed that at day 18, the cumulative amount of Hcy excreted to the culture medium by (OH)Cbl(*c*-lactam)-treated cells started to increase significantly (Figure 3a). Finally, at day 24, Hcy concentration in culture medium of the treated melanocytes was 457 ± 18% of control (Figure 3b).

### 2.4. The Effectiveness of Melanogenesis under Conditions of Cobalamin Deficiency

**Figure 4:**
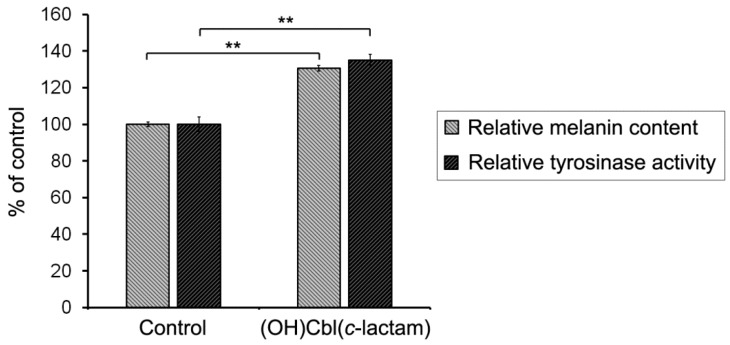
Effect of cobalamin deficiency on melanogenesis. Melanin content and tyrosinase activity in HEMn-DP cells were determined following 24 days of cell culture in medium supplemented with (OH)Cbl(*c*-lactam) in concentration of 10 μg/mL. The obtained results were calculated as the percentage of control (melanocytes cultured in parallel in standard growth medium). Data presented as the mean values of three independent experiments ± SD; asterisks denote significant differences from the control (** *p* < 0.01).

To assess the effectiveness of melanogenesis under conditions of cobalamin deficiency in vitro, we examined relative melanin content and cellular tyrosinase activity in melanocytes following 24 days of culture with (OH)Cbl(*c*-lactam) in concentration of 10 μg/mL. Treated cells were shown to have increased relative melanin content up to 131 ± 2% of the control value, while the tyrosinase activity in these cells was found to be 135 ± 3% of the value obtained for control (Figure 4).

### 2.5.Disruption of Redox Homeostasis in Cobalamin-Deficient Melanocytes

**Figure 5:**
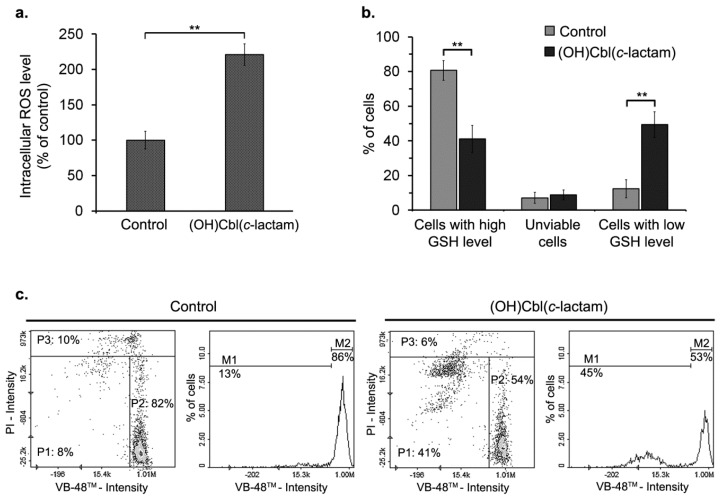
Redox homeostasis in melanocytes under conditions of vitamin B_12_ deficiency. HEMn-DP cells were cultured in medium supplemented with (OH)Cbl(*c*-lactam) in concentration 10 μg/mL for 24 days. Control melanocytes were cultured in parallel in standard growth medium. (**a**) Intracellular reactive oxygen species (ROS) level determined by H_2_DCFDA assay and calculated as the percentage of control. (**b**) Bar graphs showing the percentage of viable cells with low or high Glutathione (GSH) level and dead cells. Data presented as the mean values of three independent experiments ± SD; asterisks denote significant differences from the control (** *p* < 0.01). (**c**) Scatter plots and histograms representative for three independent experiments. Cell population was divided into three fractions: P1, M1 cells with low GSH level; P2, M2 cells with high GSH level; P3 dead cells.

Under physiological conditions, cells are in a stable state known as redox homeostasis, which is maintained by the balance between reactive oxygen species (ROS) generation and cellular antioxidants efficiency [28]. To determine whether hypocobalaminemia may affect redox homeostasis in melanocytes, we evaluated intracellular ROS level in HEMn-DP cells treated with (OH)Cbl(*c*-lactam) for 24 days. The assay with H_2_DCFDA probe revealed the increase in ROS production in cobalamin-deficient melanocytes by 120% compared to the control (Figure 5a).

Glutathione (GSH) is the most abundant low molecular weight thiol in animal cells and thus its oxidation status largely determines the thiol-disulfide status of the cell [29]. We performed cytometric analysis following fluorescent staining of cells with the thiol-group-specific dye, VitaBright 48™ (VB-48™). The data from obtained scatter plots and histograms (the representative ones were shown in Figure 5c) revealed that within the population of melanocytes treated with the cobalamin antagonist for 24 days, the percentage of cells with high GSH level decreased while the percentage of cells with low reduced thiols level increased significantly, as compared to the control (Figure 5b). There was no significant difference in the percentage of unviable (PI-positive) cells between the (OH)Cbl(*c*-lactam)-treated and control culture (Figure 5b).

## 3. Discussion

Vitamin B_12_ deficiency causes significant changes in cellular metabolism leading to various clinical symptoms [1,11]. The most common cutaneous manifestations of hypocobalaminemia include reversible skin and mucosal hyperpigmentations [12,30,31]. It was previously suggested in the literature that the dominant mechanism of these lesions is an increase in melanin synthesis rather than the defect in the pigment transfer between melanocytes and keratinocytes [13]. The current study, for the first time, provided experimental evidence supporting this hypothesis.

The aim of the first step of our work was to develop an in vitro melanocytes-based model of cobalamin deficiency by growing HEMn-DP cells in the presence of vitamin B_12_ antagonist—hydroxycobalamin(*c*-lactam). Our results showed that long-term incubation of human melanocytes with (OH)Cbl(*c*-lactam) in concentration of 10 μg/mL only slightly affected the cell viability (Figure 2a). Thus, this analogue appears to be a useful inducer of cobalamin deficiency in melanin-producing cells in vitro. We revealed, however, that long-term treatment of melanocytes with this agent caused notable reduction of cell proliferation (Figure 2b–d). In contrast to our findings, Sponne et al. [32] reported that long-term incubation with (OH)Cbl(*c*-lactam) in concentration of 10 μg/mL did not affect proliferation of rat oligodendrocytes. We suggest that this inconsistency may result from different susceptibility to cobalamin deficiency depending on the type of cell examined.

The cellular metabolic disorders due to vitamin B_12_ deficiency result in the accumulation of Hcy [1], and its export to culture medium reflects intracellular formation [33]. Therefore, by measuring extracellular Hcy concentration, the state of hypocobalaminemia in cellulo can be detected. We observed that after 18 days of cultivation with 10 μg/mL (OH)Cbl(*c*-lactam), the cumulative amount of Hcy excreted to the culture medium started to increase significantly (Figure 3a) and at day 24 the normalized value of extracellular Hcy concentration in culture medium of the treated melanocytes was 457 ± 18% of control (Figure 3b). Therefore we found that the cobalamin analogue in studied concentration is sufficient to compete with the vitamin B_12_ in the growth medium. This finding is consistent with the results presented by Sponne et al. [32]. They reported the significant increase in the Hcy concentration in oligodendrocytes medium at day 25 of cultivation with (OH)Cbl(*c*-lactam).

The observations made: (i) significant inhibition of cell proliferation (Figure 2b–d) and (ii) dramatic increase in the extracellular Hcy concentration (Figure 3a,b), indicate that cobalamin deficiency was successfully induced in human melanocytes after 24 days of culture with (OH)Cbl(*c*-lactam).

In the next step of our study we determined relative melanin content and activity of tyrosinase, the key melanogenic enzyme, in melanocytes after 24 days of incubation with (OH)Cbl(*c*-lactam). The treated melanocytes were found to have significantly increased melanin content and tyrosinase activity (Figure 4), indicating the enzyme-dependent mechanism underlying hypermelanisation. Our in vitro results concerning melanogenesis process in melanocytes under the condition of hypocobalaminemia are consistent with data from histological examinations of the specimens from patients with hyperpigmentation due to hypovitaminosis B_12_, which revealed increased melanin content in the basal layer of the epidermis [10,13].

There are some reports indicating that vitamin B_12_ deficiency induces oxidative stress in various types of cells [34,35]. Herein, we confirmed this effect in previously established melanocytes-based in vitro model. The production of ROS was determined by H_2_DCFDA staining, while cellular reduced trios status was assayed using the image cytometer. Our results revealed that ROS level in cobalamin-deficient melanocytes were extremely increased compared to the control (Figure 5a). This finding corresponds with the cytometric results which indicated that long-term treatment of melanocytes with (OH)Cbl(*c*-lactam) caused significant increase in the percentage of GSH-depleted cells with simultaneous loss of cells with high GSH level (Figure 5b,c). We found that our findings correlate with the results obtained by Sauer et al. [36]. They reported that following exposure to the cobalamin antagonist, human proximal tubule cells exhibited decreased GSH concentrations when compared to the control cells. Therefore, we suggest that the strong oxidative stress in melanocytes following the long-term incubation with cobalamin antagonist may result from the accumulation of homocysteine due to the inhibition of methionine synthase. Homocysteine is known as a potent cellular pro-oxidant since it may undergo metal-catalyzed oxidation leading to hydrogen peroxide production [37].

## 4. Materials and Methods

### 4.1. Chemicals

3,4-dihydroxy-L-phenylalanine (L-DOPA), synthetic melanin, 2′,7′-dichlorodihydrofluorescein diacetate (H_2_DCFDA), Dulbecco’s phosphate-buffered saline (DPBS) with CaCl_2_ and MgCl_2_, penicillin G, amphotericin B, SIGMAFAST™ Protease Inhibitor Coctail Tablet, and Phosphatase Inhibitor Coctail 3 were purchased from Sigma Aldrich Inc. (St. Louis, MO, USA). Neomycin sulfate was obtained from Amara (Gdynia, Poland). Medium M-254, human melanocyte growth supplement-2 (HMGS-2), and trypsin/EDTA solution were obtained from Cascade Biologics/Gibco (Carlsbad, CA, USA). Solution 5 (400 µg/mL VitaBright-48™, 500 µg/mL propidium iodide, and 1.2 µg/mL acridine orange in DMSO) was obtained from ChemoMetec (Allerod, Denmark). Cell Proliferation Reagent WST-1 was purchased from Roche (Mannheim, Germany). The remaining chemicals were purchased from POCH S.A. (Gliwice, Poland) or Sigma Aldrich. UV-visible spectra were recorded on a Jenway 7315 Spectrophotometer (Staffordshire, UK) and HITACHI Fluorescence Spectrophotometer F-7000 (Hitachi High-Tech, Tokyo, Japan).

### 4.2. Synthesis and Identification of (H_2_O)Cbl(c-lactam)Cl

(CN)Cbl(*c*-lactam) was prepared from commercially available (CN)Cbl(cyanocobalamin) according to the literature procedure described by Bonnet et al. [27]. Subsequently, it (30 mg, 22 µmol) was dissolved in H_2_O (50 mL), and Na_2_SO_3_ (1.5 g, 12 mmol) was added and the resulting mixture was stirred for 1 h at room temperature, then the solvent was evaporated under reduced pressure. The crude mixture was re-dissolved in EtOH (50 mL) and filtrated through a celite pad and evaporated under reduced pressure. The resulting solid was re-dissolved in H_2_O (18 mL) and irradiated with LED light for 1 h and then evaporated under reduced pressure. The crude was re-dissolved in EtOH (2.5 mL) and EtOH solution of aqueous hydrochloric acid (0.2 M, 2.5 mL) and stirred for 10 min followed by the addition of Et_2_O (20 mL). The precipitate was filtered through a celite pad and washed with EtOH (10 mL). The filtrate was evaporated, re-dissolved in H_2_O and chromatographed on Amerlite XAD-2 column, isocraticly, first with H_2_O (200 mL) and then with MeOH (100 mL) (Table 1). Fraction containing (H_2_O)Cbl^+^Cl^−^ was evaporated under reduced pressure, and dried in vacuo at 50 °C yielding 21 mg of aquacobalamin (16 µmol, 74%) as a red solid.

HRMS (ESI-TOF) *m*/*z* calcd for C62H86N13O14NaPCo [M + Na − H_2_O]2+: 674,768675, found 674,7673. UV/Vis (H_2_O): λ_max_ (ε) = 220 (4.78e4), 349 (2.50e4), 495 (3.97e3), 523 (8.67e3). ^1^H NMR (600 MHz, MeOH-*d*_4_) δ 7.79 (dd, *J* = 8.1, 3.8 Hz, 1H), 7.20 (s, 1H), 6.73 (s, 1H), 6.57 (s, 1H), 6.22 (s, 1H), 6.19 (d, *J* = 3.1 Hz, 1H), 4.64 (d, *J* = 9.8 Hz, 1H), 4.32 (q, *J* = 7.9, 7.2 Hz, 1H), 4.16–4.10 (m, 2H), 4.09–4.04 (m, 1H), 3.90 (d, *J* = 12.3 Hz, 1H), 3.74 (dd, *J* = 12.6, 4.1 Hz, 1H), 3.69 (dd, *J* = 14.5, 8.4 Hz, 1H), 3.45 (d, *J* = 11.0 Hz, 1H), 3.34 (s, 1H), 3.07 (dt, *J* = 11.9, 5.9 Hz, 1H), 2.80 (dq, *J* = 10.1, 4.7, 3.7 Hz, 1H), 2.78–2.69 (m, 2H), 2.69 (s, 3H), 2.65 (q, *J* = 9.0, 6.3 Hz, 7H), 2.61 (s, 2H), 2.61–2.54 (m, 2H), 2.55–2.46 (m, 1H), 2.32 (d, *J* = 5.3 Hz, 4H), 2.26 (s, 4H), 2.22–2.10 (m, 1H), 2.11–1.98 (m, 2H), 1.95 (s, 3H), 1.86 (td, *J* = 14.7, 5.1 Hz, 2H), 1.77 (ddd, *J* = 15.0, 10.5, 5.3 Hz, 1H), 1.54 (s, 3H), 1.51–1.45 (m, 1H), 1.44 (s, 3H), 1.41–1.28 (m, 1H), 1.26 (q, *J* = 6.0, 4.9 Hz, 6H), 1.21 (d, *J* = 12.1 Hz, 3H), 0.58 (s, 3H).

Data from the compound’s identification is available as supplementary material (Figure S1).

### 4.3. Cell Culture

Human epidermal melanocytes, neonatal, and dark pigmented (HEMn-DP, Cascade Biologics) were cultured according to the manufacturer’s instruction. The cells were maintained in a humidified, 5% CO_2_ incubator at 37 °C. Growth medium consisted of M-254 medium supplemented with HMGS-2, 100 U/mL penicillin G, 10 μg/mL neomycin, and 0.25 μg/mL amphotericin B. The experiments were performed using cells from passage 6–9.

### 4.4. The Induction of Vitamin B_12_ Deficiency

To achieve an experimental model of cobalamin deficiency, after preliminary experimental research, melanocytes were seeded into T-25 flask (350,000 cells/flask). The cells were cultured in medium supplemented with (OH)Cbl(*c*-lactam) in concentration of 10 μg/mL. Control cultures (melanocytes cultured in standard growth medium) were cultivated in parallel. At days 6, 12 and 18 cells were passaged to 350,000 cells/flask. The experiment was terminated on day 24 due to significant inhibition of cell growth in the treated culture. Every third day during the cultivation period, culture medium was replaced, collected and centrifuged at 2000× *g* for 20 min. The supernatants were aliquoted and stored at 20 °C until further analysis, i.e., Hcy concentration measurements.

### 4.5. Viability and Cell Count Assay

Melanocytes, when passaged at days 6, 12, 18, and 24 were analyzed according to the Chemometec’s protocol Viability and Cell Count Assay. In brief, cells were harvested by trypsinization and the samples of obtained cell suspensions were loaded into the Via1-Cassette™ and analyzed by an image cytometer NucleoCunter^®^ NC-3000™ controlled by NucleoView NC-3000™ Software (all from ChemoMetec). The cassettes contain two fluorescence probes: Acridine orange (AO) and 4′,6-diamidino-2-phenylindole (DAPI) which are known to stain the entire cell population and the non-viable cells, respectively.

### 4.6. Cell Proliferation Assay

The impact of cobalamin deficiency on cell proliferation was examined using Cell Proliferation Reagent WST-1 (Roche) according to the manufacturer’s protocol. The assay principle is based on the cleavage of the tetrazolium salt WST-1 to formazan by cellular mitochondrial dehydrogenases. The amount of formazan produced is directly proportional to the number of viable, metabolically active cells [38]. In brief, melanocytes at day 24 of culture with (OH)Cbl(*c*-lactam) were trypsinized, transferred to 96-well microplates (1 × 10^4^ cells/well) and the incubation was continued for 48 h. WST-1 reagent (20 μL/well) was added and then the plate was shaken and incubated at 37 °C for 3 h. The absorbance was measured at 440 nm (with reference wavelength of 650 nm) using the microplate reader Infinite^®^ 200 Pro, controlled by the Magellan™ software.

### 4.7. Quantitative Analysis of Extracellular Hcy Levels

Hcy is well known indicator of vitamin B_12_ deficiency in vitro because cultured cells excrete this metabolite efficiently and therefore inhibition of cobalamin-dependent metabolism increases Hcy levels in growth medium [33]. In order to verify whether the melanocytes cultured with (OH)Cbl(*c*-lactam) were cobalamin deficient, and the concentration of Hcy was measured in the medium samples collected every third day. For this purpose quantitative sandwich enzyme-linked immuno-sorbent assay (ELISA) was performed using commercially available Human Hcy ELISA Kit (Abbexa, Cambridge, UK). In brief, medium samples (50 μL/well) and then horseradish peroxidase (HRP)-conjugated detection antibody were incubated in the 96-well microplate previously pre-coated with anti-Hcy antibody. After washing, the wells were incubated with 3,3′,5,5′-tetramethylbenzidine (TMB), substrate for HRP, and then acidic stop solution was added. The absorbance of the samples was measured at 450 nm using a microplate reader Infinite^®^ 200 Pro controlled by Magellan™ software (both from Tecan, Grödig, Austria). The Hcy concentrations were calculated from the obtained standard curve.

### 4.8. Melanin Content Assay

Relative melanin content in melanocytes at day 24 of culture in medium with (OH)Cbl(*c*-lactam) was determined following the previously described method [21]. In brief, cell lysates were dissolved in NaOH (1 mol/L) at 80 °C for 1 h and centrifuged at 16,000× *g* for 20 min. The supernatants’ absorbance at 405 nm was measured using the microplate reader Infinite^®^ 200 Pro controlled by the Magellan™ software. Standard curve using synthetic melanin was prepared for each experiment. Intracellular melanin content was calculated by normalizing the melanin value with protein content (μg of melanin/mg of cellular protein) and expressed as a percentage of the control (melanocytes cultured in parallel in standard growth medium). Cellular protein concentration was determined by the use of Pierce™ BCA Protein Assay Kit (Thermo Scientific, Waltham, MA, USA) and microvolume spectrophotometer (DeNovix DS-11, USA).

### 4.9. Intracellular Tyrosinase Activity Assay

Tyrosinase activity in melanocytes was determined by the spectrophotometric assay based on the oxidation of L-DOPA to orange-red DOPAchrome, according to the previously reported method [25]. The rate of this reaction is proportional to tyrosinase activity. Cell lysates prepared from melanocytes at day 24 of culture in medium with (OH)Cbl(*c*-lactam) were clarified by centrifugation at 10,000× *g* for 5 min. After measuring protein levels and adjusting the protein concentrations to the same value, cell lysates were placed into a 96-well plate (100 μL/well) and then 40 μL of L-DOPA solution (2 mg/mL) were added to each well. Absorbance at 475 nm was measured every 10 min for 90 min at 37 °C using the microplate reader Infinite^®^ 200 Pro controlled by i-control™ software (Tecan, Austria). One unit of enzymatic activity was defined as the amount of tyrosinase which produces 1 µmol of DOPAchrome per 1 min. The enzyme activity in the samples was expressed as percentage of the control (melanocytes cultured in parallel in standard growth medium).

### 4.10. Intracellular ROS Level Assay

Reactive oxygen species (ROS) levels in melanocytes at day 24 of culture with (OH)Cbl(*c*-lactam) were estimated using the cell-permeable non-fluorescent probe H_2_DCFDA (Sigma Aldrich, D6883). After intracellular deacetylation of H_2_DCFDA to H_2_DCF, ROS oxidize H_2_DCF to generate highly fluorescent 2′,7′-dichlorofluorescein. The fluorescence intensity is correlated with the intracellular ROS level [39]. Cells were seeded at a density of 1 × 10^4^ cells/well into 96-well clear bottom black microplates and incubated in a medium with 10 μg/mL (OH)Cbl(*c*-lactam) for 48 h. Cells were then treated with H_2_DCFDA (working concentration: 20 μM) in the dark for 30 min and washed twice with DPBS to remove excess dye. The fluorescence intensity (*λ*_ex_ = 485 nm, *λ*_em_ = 530 nm) was measured using the microplate reader Infinite^®^ 200 Pro controlled by the Magellan™ software. The obtained values were normalized to the number of viable cells determined by the WST-1 assay and the results were expressed as a percentage of the control (melanocytes cultured in parallel in standard growth medium).

### 4.11. Examination of Intracellular Thiol Status

The predominant thiol in eukaryotic cells is glutathione, which is a key determinant of intracellular redox homeostasis. The overall reducing environment of the cytosol is preserved by the equilibrium between its reduced and oxidized form, abbreviated as GSH and GSSG, respectively [29]. Analysis of intracellular thiol status was performed using the NucleoCounter^®^ NC-3000™ system according to the Cell Vitality Assay protocol (ChemoMetec). In brief, the assay is based on the triple fluorescent staining using Solution 5, which contains VB-48™ and two common nucleic-acids-binding dyes. The VB-48™ reacts with intracellular thiol groups forming a fluorescent product. Acridine orange, a cell-permeable dye, stains all nucleated cells, whereas propidium iodide (PI) is capable of binding to DNA and RNA only upon the loss of cellular membrane integrity in dying, dead, and necrotic cells. Cytometric analysis of the cells fluorescence enables estimation of three subpopulations: Viable (PI-negative) cells with high GSH level, viable (PI-negative) cells with low GSH level and unviable (PI-positive) cells. After 24 days of culture in a medium with (OH)Cbl(*c*-lactam), melanocytes were harvested by trypsinization and counted using the image cytometer. Cell suspension with a density of 1 × 10^6^ cells/mL was stained with Solution 5, loaded into the 8-chamber slides (NC-Slide A8™, ChemoMetec) and analyzed with the image cytometer. By comparing scatter plots and histograms for treated melanocytes to controls (melanocytes cultured in parallel in standard growth medium) each cell population was divided into three fractions: Cells with high GSH level, cells with low GSH level, and dead cells.

### 4.12. Statistical Analysis

All data are presented as the mean values ± SD of three independent experiments in at least three repetitions. Differences between groups were analyzed by Students’ *t* test or ANOVA with Tukey’s post-hoc test using STATISTICA 13.1.336.0 (PL) Software (DELL Inc., Tulsa, OK, USA). *p* < 0.05 was considered to indicate a statistically significant difference.

## 5. Conclusions

**Figure 6:**
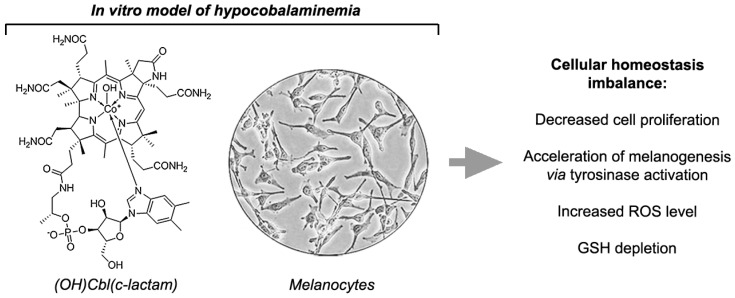
Schematic presentation of the main findings from the study.

In conclusion, our study addresses uncommon but diagnostically important symptoms of vitamin B_12_ deficiency—cutaneous pigmentary disorders. We demonstrated, for the first time, that 24-days incubation of human melanocytes with a medium containing the (OH)Cbl(*c*-lactam) in concentration of 10 μg/mL provides a powerful in vitro model for investigation whether/how hypocobalaminemia can affect homeostasis of pigment-producing cells. Our study revealed that melanocytes under conditions of hypocobalaminemia exhibit increased intracellular ROS levels, GSH depletion, and acceleration of melanogenesis via tyrosinase activation (Figure 6). Thus, we indicated important elements of the hyperpigmentation mechanism due to vitamin B_12_ deficiency, however further specific studies are needed for a deeper clarification of this phenomenon.

## Figures and Tables

**Table 1:** HPLC method.

Time (min)	H_2_O	(0.03% TFA) MeCN
0	90%	10
15	30%	70

Retention time = 5.70 min.

## Supplementary Materials

Supplementary materials can be found at http://www.mdpi.com/1422-0067/19/9/2845/s1.

## Abbreviations

AO	Acridine orange
DAPI	4′,6-diamidino-2-phenylindole
DPBS	Dulbecco’s phosphate-buffered saline
GSH	Reduced glutathione
GSSG	Glutathione disulfide
H_2_DCF	2′,7′-dichlorodihydrofluorescein
H_2_DCFDA	2′,7′-dichlorodihydrofluorescein diacetate
Hcy	Homocysteine
HEMn-DP	Human epidermal melanocytes, neonatal, dark pigmented
HMGS-2	Human melanocyte growth supplement-2
HRP	Horseradish peroxidase
L-DOPA	3,4-dihydroxy-L-phenylalanine
(OH)Cbl(*c*-lactam)	Hydroxycobalamin(*c*-lactam)
PI	Propidium iodide
ROS	Reactive oxygen species
TMB	3,3′,5,5′-tetramethylbenzidine
VB-48™	VitaBright 48™
