# Supplementary material for: Vitamin B12 Deficiency Induces Imbalance in Melanocytes Homeostasis—A Cellular Basis of Hypocobalaminemia Pigmentary Manifestations
Source: Int J Mol Sci. 2018 Sep 19;19(9):2845. doi: 10.3390/ijms19092845 (PMC6163934; doi:10.3390/ijms19092845)
Supplement: Supplementary file 1 [file ijms-19-02845-s001.pdf]

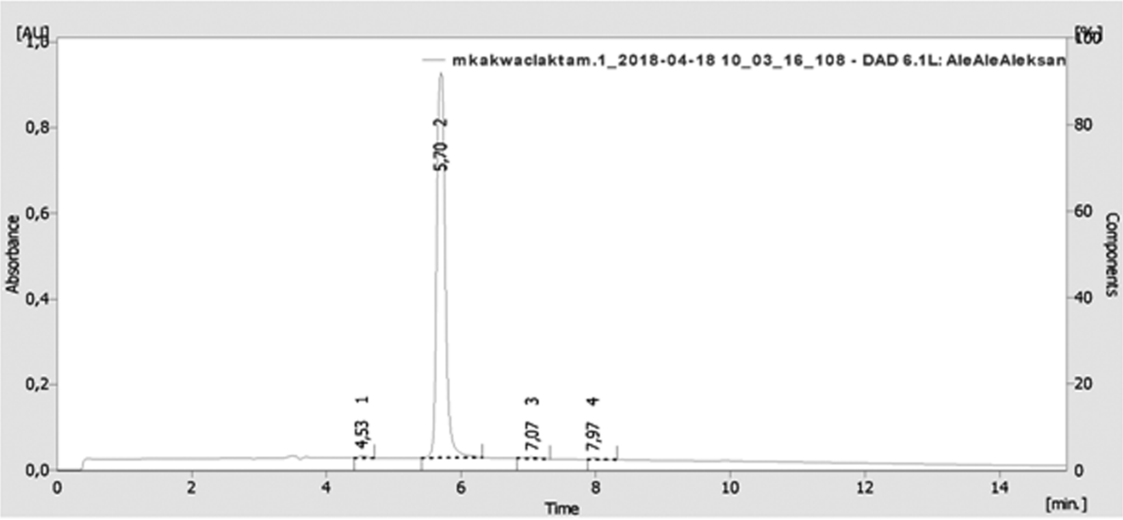

Result Table (Uncal - mkakwaclaktam.1_2018-04-18 10_03_16_108 - DAD 6.1L: AleAleAleksandra)

	Reten. Time [min]	Area [mAU.s]	Height [mAU]	Area [%]	Height [%]	W05 [min]	PDA Peak Purity	Compound Name
1	4,533	38,075	5,540	0,5	0,6	0,12	972	
2	5,700	7946,831	899,631	98,7	98,6	0,15	350	
3	7,067	44,498	3,350	0,6	0,4	0,22	910	
4	7,967	20,092	4,045	0,2	0,4	0,08	910	
	Total	8049,496	912,566	100,0	100,0			

Figure S1. Data from compound identification.
